# B-mode and contrast-enhanced ultrasound versus magnetic resonance imaging in hepatic angiomyolipoma: a case report

**DOI:** 10.1007/s40477-025-01015-w

**Published:** 2025-04-28

**Authors:** Andrea Balbinot, Sofia Maria Bakken, Nicola Venturoli, Deborah Malvi, Elisa Albertini, Maria Giulia Pirini, Antonietta D’Errico, Carla Serra

**Affiliations:** 1 Cardiovascular Medicine Unit, IRCCS Azienda Ospedaliero-Universitaria di Bologna, Bologna, Italy; 2 Department of Medical and Surgical Sciences (DIMEC), University of Bologna, Bologna, Italy; 3 Diagnostic and Therapeutic Interventional Ultrasound Unit, IRCCS Azienda Ospedaliero-Universitaria di Bologna, Bologna, Italy; 4 Pathology Unit, IRCCS Azienda Ospedaliero-Universitaria di Bologna, Bologna, Italy

**Keywords:** Hepatic angiomyolipoma, Tuberous sclerosis, CEUS, HCC, Ultrasound-guided biopsy

## Abstract

Angiomyolipoma is a solid mesenchymal tumour that usually affects the kidney. Hepatic localization of angiomyolipoma (HAML) is rare and usually asymptomatic however it presents a challenging differential diagnosis. We present the case of a 45-year-old man affected by tuberous sclerosis complex type 2 (TSC2) and an hepatic lesion suspected to be hepatocellular carcinoma on magnetic resonance but whose Bmode ultrasound and contrast-enhanced ultrasound (CEUS) findings were consistent with benignity, as confirmed by histology.

## Introduction

Angiomyolipoma is a solid mesenchymal tumour that typically affects the kidneys and contains variable proportions of dysmorphic blood vessels, smooth muscle cells and mature adipose tissue [1]. Hepatic angiomyolipoma (HAML) is rare and usually asymptomatic; therefore, it is typically detected accidentally [2, 3]. Most frequently, HAML affects non-cirrhotic livers in middle-aged women, but it is also associated with tuberous sclerosis complex (TSC) [3]. TSC is an autosomal dominant disease caused by a loss of function of genes encoding hamartin (TSC type 1) and tuberin (TSC type 2), proteins that regulate cellular growth, resulting in the development of hamartomas in numerous organ systems [4, 5]. Typically, TSC does not involve the liver; however, HAML is associated with type 2 TSC in a 5–15% of cases [6].

The differential diagnosis of HAML, particularly for hepatocellular carcinoma (HCC), can be particularly challenging because HAML can appear hyper-vascular with an early washout phase on magnetic resonance (MR) [7, 8].

## Case description

**Fig. 1:**
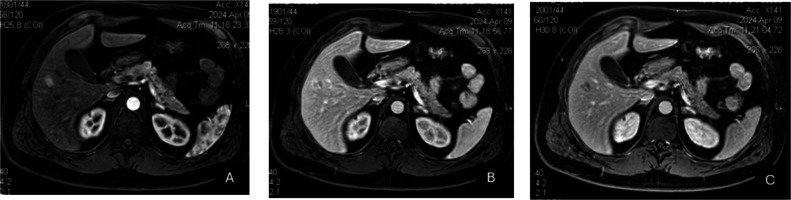
MRI aspects in arterial (**A**), venous (**B**) and tardive (**C**) phases

A 45-year-old man with no history of cirrhosis but affected by tuberous sclerosis complex type 2 (TSC 2) with cutaneous, brain, heart and kidney localization of hamartomas as well as symptoms of epilepsy since childhood, presented to our clinic because of the incidental finding of a lesion in the fifth hepatic segment on MR performed during the annual follow-up of his disease. The lesion measured 15 × 18 mm and showed a medium signal on T1 weighted images, T2 hyperintensity and arterial hyperenhancement, with early washout raising suspicion of HCC (Fig. 1).

**Fig. 2:**
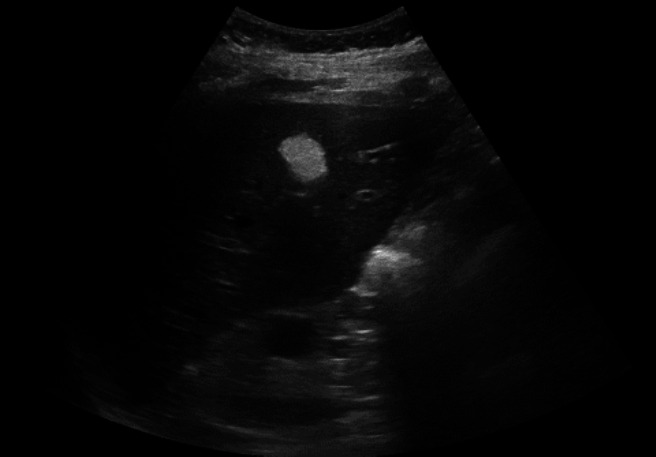
B-mode US aspect of HAML

**Fig. 3:**
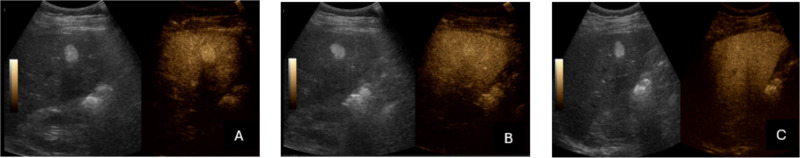
CEUS aspects in arterial (**A**), venous (**B**) and tardive (**C**) phases

We initially performed a B-mode ultrasound examination, which revealed a 17 × 13 mm bright hyperechoic lesion in the fifth hepatic segment (Fig. 2). To provide a more detailed characterization of the lesion we performed contrast enhanced ultrasound (CEUS) using the echographic contrast agent SonoVue (Bracco, Italy). CEUS showed hyper-vascular enhancement during the early arterial phase, followed by slight hyper or iso-enhancement during the venous and portal phases, compared to the normal liver parenchyma (Fig. 3).

**Fig. 4:**
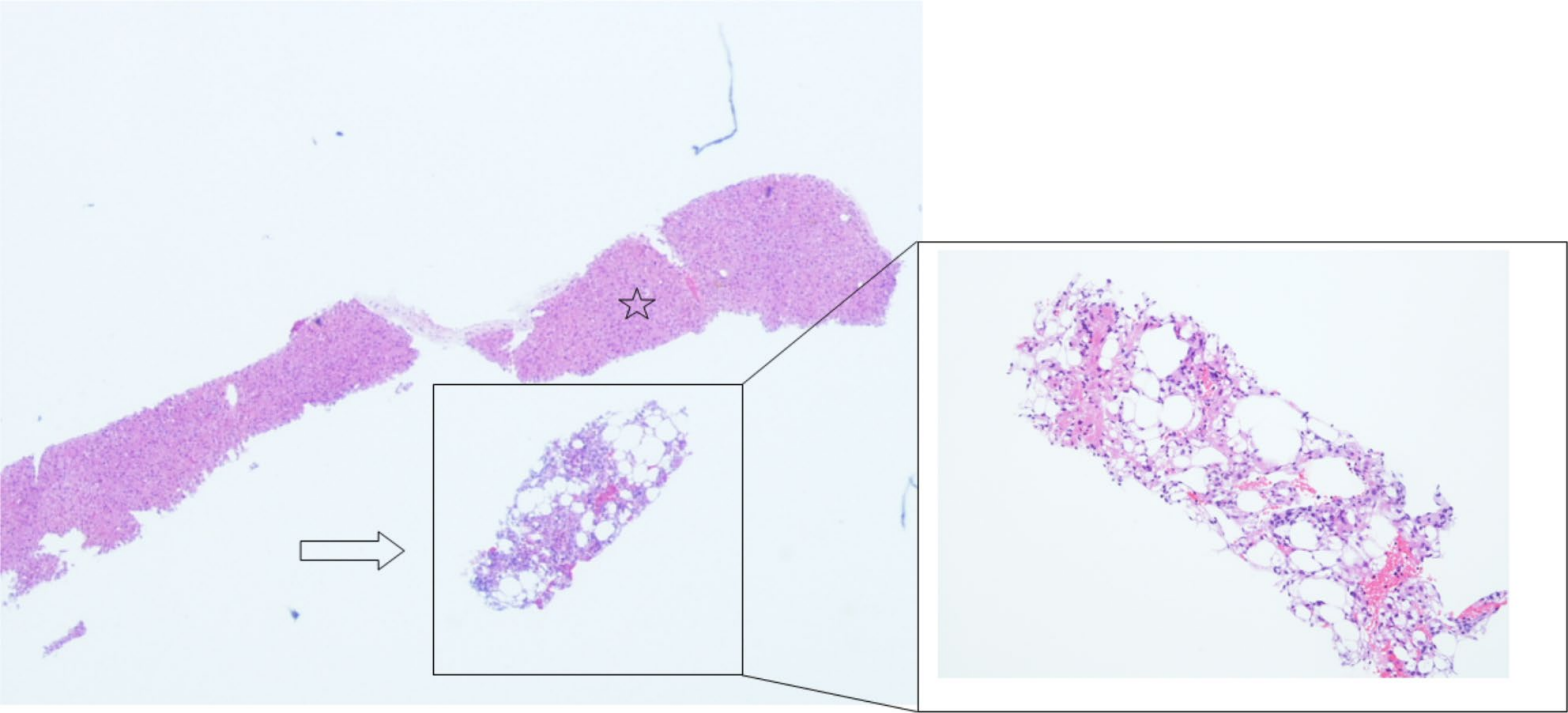
Ultrasound-guided needle core biopsy showing an adipocytic cellular lesion (arrow); adjacent benign hepatic parenchyma (star) (haematoxylin and eosin, 10 × magnification)

**Fig. 5:**
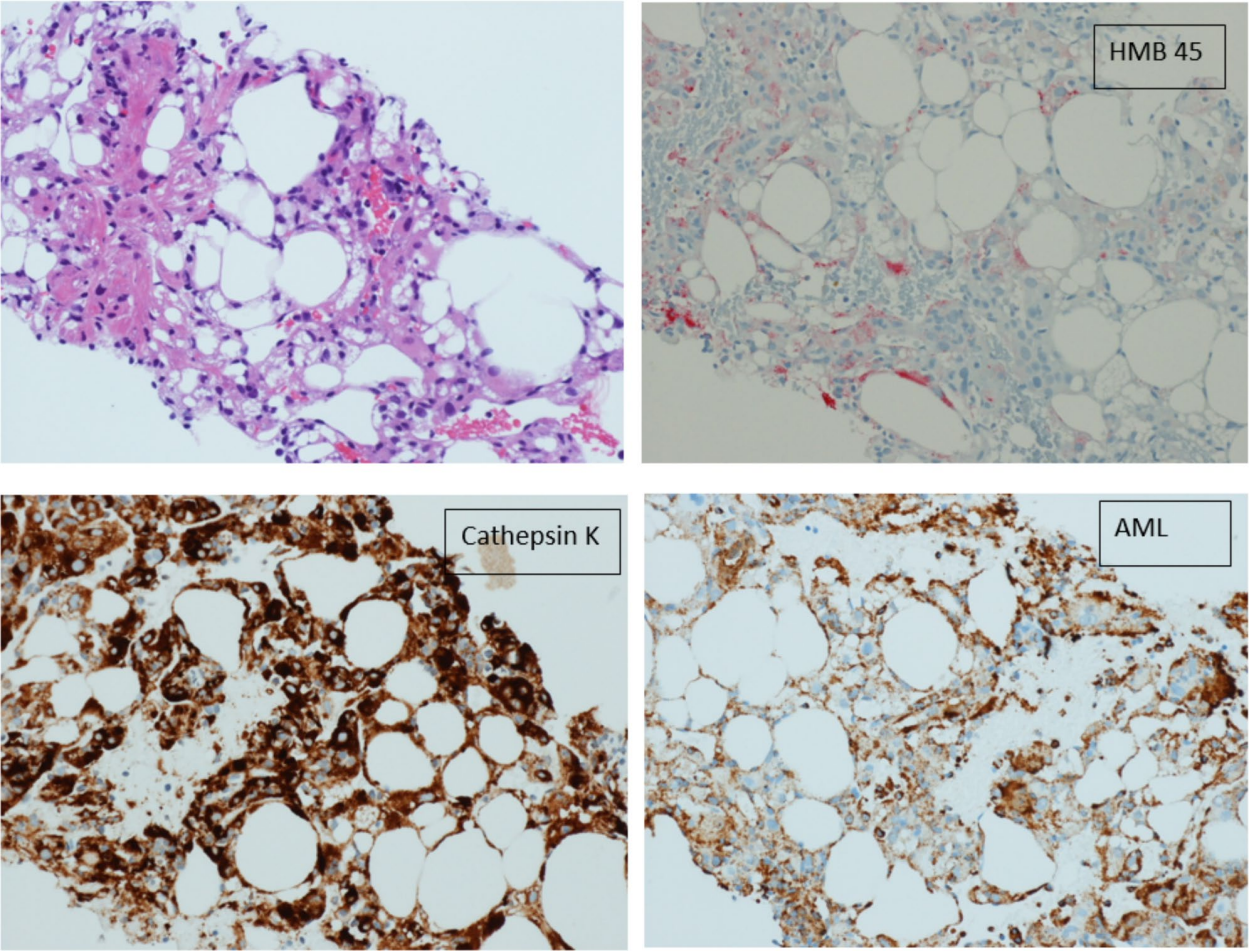
Immunostain of the lesion showing that tumor cells are positive for HMB45 (red staining) and Cathepsin K (brown staining). The spindle cells are positive for smooth muscle actin (brown staining)

Considering the patient’s clinical history, the ultrasound findings were consistent with hepatic angiomyolipoma, however, due to the rarity of this type of lesion, we performed an ultrasound-guided biopsy using an 18-gouge Tru-Cut needle. Histologically the tumor was composed of mature fat, thick-walled blood vessels and smooth muscle cells (Fig. 4). Tumour cells were positive for both smooth muscle actin, HMB 45 (Human Melanoma Black) and Cathepsin K, leading to the diagnosis of multifocal synchronous hepatic angiomyolipoma (Fig. 5), [2, 9].

## Discussion

Hepatic angiomyolipoma (HAML) is a rare hepatic lesion, with only around 600 cases described in the literature [3] As noted, in previous studies, the differential diagnosis between HAML, hepatocellular carcinoma, and other hepatic lesions is challenging, as HAMLs can exhibit contrast enhancement resembling that of malignant lesions on both magnetic resonance imaging and computed tomography [7].This behaviour is less commonly observed with CEUS, where HAML shows different quantitative contrast-enhanced parameters [10] and does not show washout in most cases (75%) [11]. The differences in contrast enhancement between HCC and HAML are likely related to both the vascular and histological characteristics of HAML, as well as the chemical properties of the different contrast media. The presence of macrophages and absence of hepatocytes are histological features of HAML that could explain these differences. [12]

Therefore, B-mode US and CEUS provide a better characterization of these lesions compared to MR, with sensitivity and specificity for distinguishing HAML from HCC ranging from approximately 66.7–100.0% [10, 13, 14]. Despite biopsy of all liver lesions, especially HCC suspicious, is not preferable, in doubtful cases, such as the one described in our study an ultrasound-guided biopsy can be safely performed. HAML is usually asymptomatic however, mass effect due to compression, and a malignant transformation potential have been reported in the literature. Due to the rarity of HAML, its optimal clinical management remains controversial [15]. Considering the histological diagnosis, the small size of the lesions and the absence of signs of hepatic cirrhosis, we preferred for conservative management with close follow-up every 6 months.

## Conclusions

B-mode ultrasound and CEUS, along with the option of performing an ultrasound-guided biopsy in doubtful cases, could be superior diagnostic tool for differentiating HAML from HCC. Furthermore, when conservative management is preferred, they also offer an effective follow-up strategy compared to MR and CT.

## Data Availability

The data that support the findings of this study are available from the corresponding author upon reasonable request.
